# Hybrid paper sheets with improved barrier properties

**DOI:** 10.3906/kim-2101-43

**Published:** 2021-08-27

**Authors:** Çağla SÖZ

**Affiliations:** 1 Department of Material Science and Technologies, Faculty of Science, Turkish-German University, İstanbul Turkey

**Keywords:** barrier properties, Cobb value, hybrid paper sheets, packaging, roughness, superhydrophobic coatings, tensile index, wetting

## Abstract

Hybrid paper sheets were prepared by applying a thin coating layer of cross-linked polydimethylsiloxane (PDMS) and inorganic particles onto Whatman Grade 1 filter paper substrates. Several coatings with different inorganic particle contents and types were applied onto the paper substrates to investigate the effect of the variation in the coating formulation on the (i) wetting, (ii) water barrier properties, (iii) air barrier properties, (iv) surface roughness, and (v) mechanical properties of the samples. It was revealed that the superhydrophobic hybrid paper sheets with significantly low air permeability and high water barrier properties could be prepared which is an indication that the method proposed can be used for the preparation of packaging materials.

## 1. Introduction

Hybrid paper sheets are paper-based composite materials composed of organic and inorganic components, often prepared by modifying the surface of the paper substrate with a coating composed of polymeric and inorganic components. Paper has gained great interest as the substrate for preparation of hybrid materials because it has many advantages: Paper is a nonwoven material composed of cellulose fibers, with a highly optimized industrial production process and a simple production path [1]. Paper is a promising starting material with its unique properties such as high porosity and low density, especially for membrane type systems. In addition, the large surface area of ​ paper can be further expanded by grinding processes according to the desired application [2]. However, due to the presence of a large number of hydroxyl groups, the paper surface can be easily modified by covalent bonding of various chemicals [2,3]. Paper is thin and flexible, does not break when bent, and shows almost no thermal expansion [3]. Paper is also a biogenic material [4]. Cellulose, the main component of paper, is the most abundant polysaccharide in nature and is renewable, inexpensive, and biodegradable. It can be obtained from plants (cotton linters, bamboo, flax) as well as wood (softwoods such as spruce or pine, and hardwoods such as poplar, birch or beech) [5]. The fact that cellulose is available from a wide variety of natural sources allows the preparation of papers with quite different morphological, topographic, and chemical properties. In particular, its porous structure and the fact that both the chemistry and topography of its surface can be easily modified make paper an extremely suitable substrate for designing different hybrid materials that interact with water, water vapor, and air as desired.

Because of all these advantages summarized above, paper is used as substrate in many highly sophisticated studies. Studies on hybrid paper materials, which are aimed to serve different purposes such as smart membranes, sensors, electronic applications, with the addition of a wide variety of filling materials, are common [6,7]. Also, scientific studies on paper materials considered as packaging materials or shopping bags occupy a large place in the literature [8–11].

To prepare new and robust alternatives to the samples seen in literature, superhydrophobic hybrid paper sheets having durable superhydrophobic surfaces were prepared, which possessed significant air and water barrier properties. This study on hybrid paper sheets can be regarded as the continuation of the work carried out in 2018, in which covalent bonding of inorganic particles in the coating layer to the paper substrate surface were achieved via polydimethylsiloxane (PDMS) chains so that new hybrid paper sheets with both high chemical and mechanical stability were obtained [12]. Here, the inorganic filler type and content in coating formulation was changed to investigate the effect of this change on (i) wetting, (ii) water barrier properties, (iii) air barrier properties, (iv) surface roughness, and (v) mechanical properties of the hybrid paper sheets.

## 2. Materials and methods

### 2.1. Materials

Trimethylsiloxy-terminated PDMS (silicone oil) with a molecular weight of 5970 g/mol was purchased from Gelest (Morrisville PA, USA). Glass spheres (GS), montmorillonite (MMT), and kaolin (K) were purchased from Sigma Aldrich. Aeroperl 300 Pharma colloidal silica (aero) was kindly supplied by Evonik (Istanbul, Turkey) via Marmara Ecza (Istanbul, Turkey). HDK N20 fumed silica was kindly supplied by Wacker (Istanbul, Turkey) via IMCD Group (Istanbul, Turkey). Whatman Grade 1 filter paper (WFP) was purchased from General Electric (Turkey). Reagent grade tetrahydrofuran (THF) and isoproponal (IPA) were purchased from Merck (Germany) and used as received. 

### 2.2. Preparation of hybrid paper sheets 

Sample preparation procedure was reported in previous study [12]. Briefly, PDMS was dissolved in THF so that a dilute solution of 0.7% PDMS by weight was obtained, into which three types of filler particles were added, each equivalent in weight to PDMS, so that weight of the filling materials in the mixture corresponded to 3 times the weight of PDMS (Table 1). The dispersion mixture was mixed with a magnetic stirrer and then in an ultrasonic bath and transferred to the tank of the spray gun without delay. The dispersion mixture was applied onto Whatman Grade 1 with a spray gun while the distance between the spray gun nozzle and the paper substrate was kept constant. Afterwards, the samples were left in the hood overnight for the evaporation of the THF solvent. Samples were then heat-treated at 120 °C for 36 h. After the heat treatment step, samples were left to cool to 23 °C. Soxhlet extraction with THF was performed to get rid of the excess/unreacted PDMS.

**Table 1:** Sample sets prepared in this study.

#	Sample code
1	WFP (control)
2	PDMS/GS/aero/N20
3	PDMS/MMT/aero/N20
4	PDMS/K/aero/N20
5	PDMS/MMT/GS/N20
6	PDMS/K/GS/N20
7	PDMS/MMT/K/N20

At least 10 samples were prepared for each of the above listed sample sets. It is important to note that the PDMS/GS/aero/N20 sample has a similar composition with those of the samples prepared in our previous study. The aim of preparing PDMS/GS/aero/N20 is to be able to compare the properties of the 6 above mentioned sample sets having different coating formulations with each other and with WFP control.

### 2.3. Characterization

#### 2.3.1. Dynamic and static contact angle (CA) measurements

Static and dynamic contact angles of the samples were measured with Dataphysics OCA 15 goniometer equipped with SCA 20_U software. Before the tests, the samples were conditioned for 24 h at 23 ± 2 °C and 50 ± 5% relative humidity. Static contact angle (CA) measurements were performed by the sessile drop method. Ten **μL** of triple distilled deionized water was dropped onto the sample surface, and the CA was measured at a temperature of 23 **±** 2 **°C.** Ten measurements were performed for each surface so that an average static water CA could be determined. Dynamic contact angles were measured with the embedding needle method. First, 1 **μL** of distilled and deionized water droplet was injected at a speed of 0.2 μL/min from the needle tip in a controlled manner and touched the surface. Then, the volume of the droplet was increased to 5 **μL** at a speed of 0.2 **μL** /min. The volume of the 5 μL drop was increased to 25 **μL** with a speed of 0.2 **μL** /min without retracting the needle, and the highest CA value observed during this volume increase was recorded as the advancing water contact angle (θ_adv_). Subsequently, the volume of the water drop was reduced to 5 **μL** with a speed of 0.2 **μL** /min without retracting the needle, and the lowest CA value measured was recorded as the receding water contact angle (θ_rec_). The contact angle hysteresis (CAH) value was determined as θ_adv_– **θ**_rec_ [13]. 

#### 2.3.2. Water imbibition tests 

Samples with sizes of of 2 × 2 cm^2^ were conditioned by waiting for 24 h in the conditioning chamber adjusted to 23 ± 2 °C temperature and 50 ± 5% relative humidity. After conditioning, the samples were weighed and put in 20 mL of distilled and deionized water at 23 ± 2 °C and 50 ± 5% relative humidity. The soaking duration the samples in water was kept at 24 h, optimized by previous studies. At the end of 24 h, the samples removed, excess water on sample surfaces were taken with a clean cotton swab and then the weight was measured again. The recorded weight gain gave the amount of water absorbed by the sample and the water absorption capacity of the sample was calculated according to Eq. (1). This test was repeated at least 3 times for each sample. 

(1)% water absorption by weight = final weight - initial weightinitial weightx 100

#### 2.3.3. Determination of air permeance 

Before the tests, the samples were conditioned for 24 h at 23 ± 2 °C and 50 ± 5% relative humidity. For the determination of air permeance, L&W Air Permeance Tester was used and the measurements were taken in accordance with the Tappi T 460 om-88 standard. At least 5 measurements were taken for each sample at room temperature.

#### 2.3.4. Determination of Bendsten roughness

Before the tests, the samples were conditioned for 24 h at 23 ± 2 °C and 50 ± 5% relative humidity. For the determination of the Bendtsen roughnesses of the samples, measurements were made by using the Bendtsen method according to the Tappi T 479 om-91 standard using the L&W Bendtsen Tester. At least 5 measurements were taken for each sample at room temperature.

#### 2.3.5. Cobb values

Before the tests, the samples were conditioned for 24 h at 23 ± 2 °C and 50 ± 5% relative humidity. The Cobb test was carried out in accordance with the Tappi T 441 standard using a 10 kg stainless steel metal cylinder and the sample holder. Briefly, 100 mL of distilled water was poured into the chamber of the sample holder under which a pre-weighed paper-based sample fixed with the modified surface facing water. Afterwards, water in the reservoir was quickly emptied. In order to remove the excess water on the paper sample, a 10 kg stainless steel cylinder was passed over the sample with an absorbent paper placed on top of the sample surface. Finally, the sample weight was measured again. Cobb values of the sample were calculated with Eq. (2). At least 5 measurements were taken for each sample at room temperature. 

(2)Cobb value (g/m2)=(weight of wet sample - weight of dry sample)sample area (0.01 m2)

#### 2.3.6. Tensile tests

Dry and wet tensile strength values of the sample strips were determined by using a Zwick 4831 Z2.5 Stress-Strain Instrument. For measurement of the dry tensile strength values, test strip widths and lengts were kept at 12.5 mm and 50 mm, respectively. For measurement of the wet tensile strength values, on the other hand, test strip widths and lengts were kept at 12.5 mm and 90 mm, respectively. Dry and wet paper strips were clamped to the clamps of the instrument, and the measrurements were performed according to the TS EN ISO 1924-2 and TS 5163 ISO 3781 (Finch method) testing standards, respectively. Dry and wet tensile strengths were determined both for the mashine direction (MD) and the cross direction (CD) of the samples. At least 5 measurements were performed for each sample. The tensile strength values were divided to the average grammage value of each sample to achieve the tensile index values, as shown in Eq. (3).

Tensile index = tensile strength [N/m]/grammage [g/m^2^] = tensile index [Nm/g]. (3)

The relative wet strength was calculated by using the the dry and wet tensile stregth values according to Eq. 4:

(4)Rel. wet stregth in % =wet tensile strengthdry tensile strengthx 100

#### 2.3.7. Surface analysis with scanning electron microscope (SEM)

SEM images were taken with a Zeiss Evo MA10 scanning electron microscope. Prior to the SEM studies, the samples were coated with a thin gold layer of around 12 nm to prevent/minimize charging on the surface. 

#### 2.3.8. FT-IR analysis

Attenuated total reflection fourier transform infrared (ATR-FTIR) measurements were performed by using a Perkin-Elmer FT-IR: ATR spectrometer equipped with a flat diamond plate. The spectra of solvent cast and dried films were collected with a resolution of 4 cm^–1^ and 32 scans were obtained for each spectrum.

## 3. Results and discusssion

### 3.1. Preparation of the hybrid paper sheets

In this study Whatman Grade 1 (WFP) cotton linters paper was used as the paper substrate because it consists of pure cellulose fibers and thus has the highest number of reactive hydroxyl groups on its surface. As mentioned before, the study aims to understand the effect of the coating formulation on the (i) wetting, (ii) water barrier properties, (iii) air barrier properties, (iv) surface roughness, and (v) mechanical properties of the hybrid paper sheets. The studies were conducted using WFP first, so that the knowledge gained can be applied for coating of different papers such as softwood Kraft paper in the future, which may have specific applications as packaging papers. 

As mentioned in the previous study, it was inspired by the studies of Botter et al., Soares et al., and Krumpfer and McCarthy so that a highly practical method was developed to prepare hybrid paper sheets [14–16]. In the aforementioned studies, it was proven that trimethylsilyl-terminated polydimethylsiloxane (PDMS) chains, also known as silicone oil and considered nonreactive, can be covalently bonded to various inorganic surfaces [14–16]. To explain the covalent bonding, two reaction mechanisms were hypothesized: (i) the hydrolysis of PDMS followed by condensation with a surface silanol in the presence of a minor amount of water and (ii) the direct or acid-catalyzed silanolysis of PDMS by a surface silanol [16]. These mechanisms, which have been suggested only for the modification of inorganic surfaces, have been seen by us as a suitable method for modifying organic paper surfaces, since both the cellulose fibers forming the paper and the inorganic particles selected within the scope of the study naturally show hydrophilic and slightly acidic properties. So, mixtures of inorganic particles with trimethylsilyl terminated PDMS were applied to the paper surfaces. After the drying and heat treatment stages, it was determined that PDMS was bonded both to the surface of the cellulose fibers and the inorganic particles in the mixture acting as bridges between those besides hydrophobizing the surface. In this study, 6 sample sets were prepared according to the same sample preparation procedure (Table 1) and investigated. The sample sets were hybrid paper sheets achieved through the coating application of the mixture composed of PDMS and inorganic particles with different inorganic particle contents. WFP was selected as the control sample of the study and its properties were also tabulated. In literature, usually platelet shaped clays [17–22], and colloidal or agglomerated particles [23–30] are used to enhance the barrier properties of the coated substrates against water or air. Since the aim of the study was to enhance the barrier properties of the samples as much as possible, no sample with just PDMS coating layer was tabulated. Moreover, nonwettability of hybrid paper sheets was another important goal to achieve, which has a significant impact on water barrier properties. It is already known in literature that, while only the polymer coating is sufficient to make the paper surface hydrophobic [30], the addition of fillers is necessary in obtaining nonwettable, superhydrophobic paper surfaces [23–28]. So, mixtures of PDMS and inorganic particles instead of pure PDMS were used for surface modification of paper substrates. 

The inorganic fillers used in the study are glass spheres, colloidal and fumed silica particles, and clays. Montmorillonite clay nanoparticles with smectite structure consist of stacked and plate-like silica layers with a high surface area (700–800 m^2^/g) and a thickness of about 1 nm. Having a 2:1 phyllosilicate (an Al-O octahedral layer sandwiched between two Si-O tetrahedral layers), these particles have intrinsic hydrophilicity [6,8,9,11,17,18]. Kaolin, on the other hand, is a 1: 1 phyllosilicate clay composed of an octahedral gibbsite layer and a tetrahedral silicon oxide layer. Kaolin, which is abundant in soil and sedimentary rocks, is widely used in industry [19,20]. Both fillers have a high aspect ratio and large surface area to interact with polymer chains [10]. Incorporation of kaolin and montmorillonite to polymeric materials to coat paper surfaces is widely studied in literature, especially in preparation of novel packaging materials [7–10,21,22]. Addition of colloidal or fumed silica-based polymeric nanocomposite coatings on paper surfaces is also another frequently preferred method in literature [23]. For example, Gao et al., Tasleem et al., Musikavanhu et al., Shu et al., Zhang et al., and Sriram and Kumar’s studies on hydrophobic and superhydrophobic paper-based materials are just some of the studies published on the subject published in recent years. [23–29]. In our study, effect of filler content on the final properties of the hydrid sheets was investigated.

### 3.2. Wetting properties 

Within the scope of this study, wetting characteristics of hybrid paper sheet with water play an important role. Measuring contact angles, an optical method that has a history of more than 200 years, is considered to be the gold standard in characterizing the wetting characteristics of various surfaces [31]. If the adhesion force of the water droplet is much greater than the surface tension force of the surface under examination, the water drop wets the surface, but if the adhesion force is much smaller than the surface tension force, the water droplet on the surface takes a spherical shape. The easiest way to examine the relative magnitudes of these forces is to measure the static water contact angle (CA) between the the tangents drawn to the liquid-gas interface and liquid-solid interfaces intersecting each other at the three-phase contact line. Hydrophilic surfaces have static water contact angles smaller than 90 ° whereas hydrophobic ones greater than 90 ° and superhydrophobic surfaces greater than 150 ° [32]. Superhydrophobic surfaces are also expected to have contact angle hysteresis (CAH) or tilt angle values below 10 ° [13]. CA values describe the equilibrium state of water dropped on surfaces. However, each surface has a series of semi-stable contact angles and the static water contact opener falls within this range of semi-stable values. Therefore, the minimum and maximum contact angle values of the semi-stable range, called receding and advancing contact angles (θ_rec _and θ_adv_, respectively), need to be measured [13]. These measurements are named as dynamic measurements. The CAH value is defined as the difference between the advancing and receding contact angles (or the difference between the cosine values of these angles). The higher the water contact angle hysteresis value, the less slippery is the surface for the droplet [13].

Because of being very hydrophilic, WFP absorbed the water droplet immediately resulting in CA values of 0 °. WFP substrates coated with a PDMS layer without inorganic particles had also CA values around 0 °, which revealed that the thin PDMS coating alone was not sufficient to cover and hydrophobize the whole porous surface of WFP so that the water droplets could be adsorbed by the porous paper material. On the other hand, the coated hybrid sheets having WFP as the substrate had all superhydrophobic properties with similar CA Values above 150 ° and CAH values below 10 °, as shown in Table 2. Table 2 also contains information about the water imbibition values of the samples. The coated hybrid sheets had lower water imbibition values than that of the WFP, as expected. There is an approximately 32% decrease in water imbibition for hybrid paper sheets with respect to the WFP. And, it should be kept in mind that only one side of the samples were coated. And, it is clear that the superhydrophobic coating decreases the water imbibition capacity of the samples. 

**Table 2:** CA, CAH and water imbibition values of the control WFP and the hybrid sheets.

Sample code	CA (°)	CAH (°)	Water imbibition (wt %)
WFP (control)	0	-	150.8 ± 2.2
PDMS/GS/aero/N20	161.9 ± 1.5	5.4 ± 1.0	102.3 ± 1.2
PDMS/MMT/aero/N20	163.7 ± 1.9	5.7 ± 1.1	114.7 ± 9.9
PDMS/K/aero/N20	162.3 ± 1.7	7.6 ± 1.7	125.1 ± 4.6
PDMS/MMT/GS/N20	160.1 ± 0.1	7.6 ± 0.4	114.4 ± 18.6
PDMS/K/GS/N20	160.5 ± 1.7	8.5 ± 0.3	117.3 ± 0.4
PDMS/MMT/K/N20	163.4 ± 2.0	3.4 ± 0.7	101.9 ± 5.9

### 3.3. Barrier properties 

Besides being nonwetting with high CA and low CAH values, hybrid paper sheets should also possess barrier properties against water and air for being considered as potential candidates for packaging materials [33–37]. In literature, the resistancy of these materials towards various environmental factors like gases, temperature, moisture, or grease is frequently reported [33–37]. But, the resistancy against water and air are the two most crucial factors in determining the barrier performance of a paper based material [34–36]. Usually, a polymeric composite coating is applied onto the paper or paperboard sustrate to achieve the desired barrier properties [33,34,37]. To get an idea about the barrier performances of the hybrid paper sheets, their air permeance, Bendtsen roughness and water absorption values were measured in accordance with the globally accepted Tappi standards. Air permeace tests reveal the barrier properties of paper-based samples against compressed air and performed with the L&W Air Permeance Tester. Paper roughness analyses, on the other hand, are traditionally performed with the pneumatic Bendtsen Instrument. By roughness of the paper substrate, the degree of unevenness or irregularity over the surface is meant. The analysis is relatively easy to apply and give stable results, but does not make a roughness measurement, gives rather an idea about it in an indirect fashion [38]. For the measurement, the test piece is first clamped between a flat glass plate and a circular metal ring, the ring is pressed onto the paper surface, and the air leak between the ring and the paper or paper-based substrate is detected in unit time, so that a qualitative result about the roughness is achieved [39,40]. The greater the air leak, the rougher the surface [38]. Cobb tests are used to calculate the mass of water absorbed by one square meter of paper (based) sample at a given time and is a well known measure for water barrier properties of paper and paper based materials. [41–43]. Air permeance and roughness values (mL/min) and Cobb test results (g/m^2^) of the samples are given in Table 3.

**Table 3:** Air permeance and roughness values (mL/min) and Cobb test results (g/m2) of the hybrid paper sheets.

Sample code	Air permeance(mL/min)	Bendsten surfaceroughness (mL/min)	Cobb value (g/m2)
WFP	3215.0 ± 63.6	1702.0 ± 43.8	220.2 ± 0.3
PDMS/GS/aero/N20	2885.0 ± 17.1	726.5 ± 53.0	47.8 ± 15.2
PDMS/MMT/aero/N20	2620.0 ± 56.8	777.0 ± 31.6	24.1 ± 7.1
PDMS/K/aero/N20	1500.0 ± 50.9	823.7 ± 48.7	20.8 ± 0.6
PDMS/MMT/GS/N20	2073.3 ± 18.5	952.0 ± 76.4	26.3 ± 3.1
PDMS/K/GS/N20	1493.3 ± 16.6	771.0 ± 132.9	16.5 ± 0.6
PDMS/MMT/K/N20	498.5 ± 85.6	1338.5 ± 29.0	19.7 ± 2.9

Table 3 reveals that the filter paper has the highest air permeance, as expected. Hybrid paper sheets with a coating layer on top show lower air permeance values than the filter paper. The significant differences between the values are due the difference in filler types in the coating formulation including glass spheres, colloidal silica, montmorillonite, and kaolin. Each coating contains the same ratio of PDMS and N20 fumed silica and was prepared onto the WFP substrate. Therefore, the effect of PDMS, N20, and WFP onto the barrier properties cannot be considered within this study.

PDMS/GS/aero/N20 samples showed lower air permeability than that of filter WFP. When the glass spheres (GS) in the coating are replaced with montmorillonite (MMT) or kaolin (K), respectively (see PDMS/MMT/aero/N20 and PDMS/K/aero/N20), the air permeability gradually decreased. This shows that the platelet structured montmorillonite and kaolin fillers are more effective in reducing the air permeability of hybrid sheets than spherical glass spheres. When the colloidal silica in the coating formulation was replaced with montmorillonite or kaolin fillers (see PDMS/MMT/GS/N20 and PDMS/K/GS/N20), a similar decrease in air permeability occured. And, kaolin was found to be more effective than montmorillonite filler in reducing the air permeability of the hybrid paper sheets. The least air permeance was shown by the PDMS/MMT/K/N20 samples with the highest amount of platelet-shaped fillers, which are known to have the ability to decrease gas permeability [7–10,21,22]. It can be seen that the hybrid paper sheets listed above show different air permeance properties due to the effect of the different fillers they contain in their coating layer.

The Bendsten roughness values of hybrid sheets in Table 3 are lower than that of the WFP. The effect of filler type on the roughness values can be summarized as follows: PDMS/GS/aero/N20 samples have the lowest Bendsten roughness values among hybrid paper sheets. When the glass spheres (GS) in the coating were replaced with montmorillonite (MMT) or kaolin (K), respectively (see PDMS/MMT/aero/N20 and PDMS/K/aero/N20), Bendsten roughness values gradually increased. When colloidal silica in the coating content was replaced with montmorillonite or kaolin fillers, respectively (See PDMS/MMT/GS/N20 and PDMS/K/GS/N20), a similar increase in Bendsten roughness values was seen. The highest Bendsten roughness values among the hybrid paper sheets were seen in PDMS/MMT/K/N20 samples. The data revealed that the addition of platelet shaped inorganic fillers into the coating layer of hybrid paper sheets was more effective in increasing the Bendsten roughness values than the addition of colloidal or spherical inorganic particles.

The Cobb values of hybrid paper sheets reveal the significant increase in water barrier properties of these superhydrophobic samples with respect to that of WFP. Although CA and CAH values are significant scientific data that give an idea about the interaction of hybrid paper sheet surfaces with water, the Cobb test is mainly used in the paper industry to determine the interaction/barrier properties of paper-based samples with/against water [42]. As shown in Table 3, WFP has a Cobb value of 220.2 ± 0.3 g/m^2^, while Cobb values of the superhydrophobic hybrid paper sheets remain all below 50 g/m^2^. So, the coating layer decreases the water barrier properties of the sheets over 75%. It is reported in literature, that samples with water absorption values of 50 g/m^2^ or below are accepted as samples with high water barrier properties in paper industry [42,43]. The PDMS/GS/aero/N20 samples had the highest Cobb value. When glass spheres (GS) in the coating were replaced with montmorillonite (MMT) or kaolin (K) (See PDMS/MMT/aero/N20 and PDMS/K/aero/N20), a decrease in Cobb values was observed. When the colloidal silica in the coating content is replaced with montmorillonite or kaolin fillers (PDMS/MMT/GS/N20 and PDMS/K/GS/N20), a similar decrease is observed in Cobb values. All in all, the data reveals that (i) the platelet-shaped fillers in the coating layer increased the water barrier properties of the samples, (ii) kaolin was more effective than montmorillonite in increasing the water barrier properties. 

### 3.4. Mechanical properties of hybrid paper sheets 

In this study two sets of hydrid paper sheets having the highest difference between the barrier properties, PDMS/GS/aero/N20 and PDMS/MMT/K/N20, were selected for tensile tests in dry and wet states. Tensile test results of the WFP were also recorded and compared with those of the hybrid paper sheets. Meauring the tensile indices is a widely preferred route for the analysis of the mechanical strength of paper [44,45]. Since commercial paper substrates are used and modified in that study, analyses were made both in mashine and cross directions (MD and CD). The dry and wet tensile indices of the abovementioned samples are listed in Table 4.

**Table 4:** Tensile test results.

Sample code	Dry tensile index (MD) (Nm/g)	Dry tensile index (CD) (Nm/g)	Wet tensile index (MD) (Nm/g)	Wet tensile index (CD) (Nm/g)
WFP	30.68 ± 1.41	20.06 ± 0.62	1.17 ± 0.10	0.66 ± 0.02
PDMS/GS/aero/N20	23.31 ± 0.65	19.51 ± 1.28	2.49 ± 0.35	1.73 ± 0.37
PDMS/MMT/K/N20	23.01 ± 1.69	18.77 ± 1.20	2.97 ± 0.63	2.32 ± 0.17

As revealed in Table 4, coating application slightly decreased the dry tensile index values. However, the wet tensile indices of the hybrid paper sheets are much higher than those of WFP. For PDMS/GS/aero/N20, there are 112.8% and 162.1% increase in tensile indices in MD and CD with respect to those of WFP, respectively. The increase is more pronounced for PDM/MMT/K/N20: increases of 153.9% and 251.5 % were recorded for tensile indices in MD and CD, respectively. So, the superhydrophobic coating seems to protect the integrity of the fibers in wet state. The relative wet strength values of the samples were also calculated by using Eq. (4). While the relative wet stregth value of WFP is 3.7% and 3.4% in MD and CD, an almost 3-fold increase was detected and these values became 10.5% (MD) and 9.8% (CD) for PDMS/aero/GS/N20. The increase was higher for PDMS/MMT/K/N20 sheets with relative strength values of 12.9% (MD) and 9.84% (CD). It can be concluded that the superhydrophobicity of the coating and enhanced water barrier properties of PDMS/MMT/K/N20 sheets have a positive impact on their wet tensile test values.

### 3.5. SEM investigations of the hybrid paper sheets 

**Figure 1:**
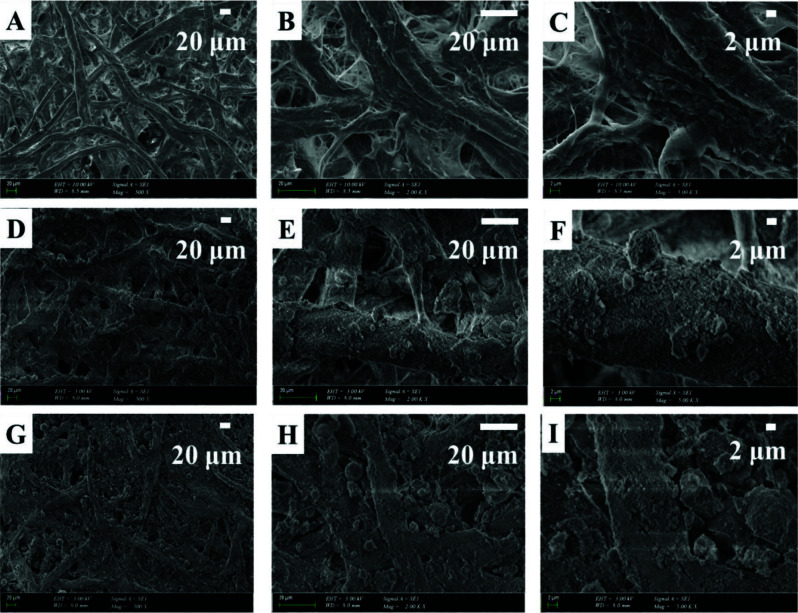
SEM images of WFP (A, B, and C), PDMS/GS/aero/N20 (D, E, and F), and PDMS/MMT/K/N20 (G, H, and I) surfaces with magnification levels of 500X, 2000X, and 5000X, respectively.

SEM studies were performed to reveal the significant differences in surface topographies of WFP and hybrid paper sheets. Again, WFP, PDMS/GS/aero/N20, and PDMS/MMT/K/N20 were selected for SEM investigations, the images of which are given in Figure 1 at different magnification levels.

The characteristic fibrillar morphology of WFP can be clearly seen in Figures 1A, B, and C. PDMS/GS/aero/N20, on the other hand, had a composite coating on the paper substrate, so that the fibrillar structures were significantly embedded into the coating layer. There was a dense distribution of filler particles with different size ranges on the hybrid paper sheet surface so that a hierarchical topography of micro- and nanosized structures occured which highly resembled that of a lotus leaf surface and resulted in the superhydrophobic behavior of the sample surface [46]. Although platelet-shaped montmorillonite and kaolin was used in its coating formulation of PDMS/MMT/K/N20 had a similar degree of surface coverage and hierarchical pattern of surface protrusions resulting in superhydrophobicity of the surface. 

### 3.6 FT-IR studies 

FTIR spectroscopy was applied in order to understand the presence and nature of possible interactions between paper substrate surface and PDMS in the coating layer. The FT-IR studies can be though as a continuation of the Raman studies published previously, which were performed for monitoring the covalent attachment between PDMS and cellulose-based WFP substrate underneath [12]. Briefly, model compounds of microcrystalline cellulose (MCC) and PDMS were mixed and heat treated. Raman spectra of pure PDMS, MCC, and model compound of MCC/PDMS were taken and additional peaks at 1161 cm^−1^ and 806 cm^−1^ were detected in Raman spectrum of model compound which can be attributed to the freshly formed Si-O-C asymmetric and asymmetric stretching peaks, respectively [47]. 

**Figure 2:**
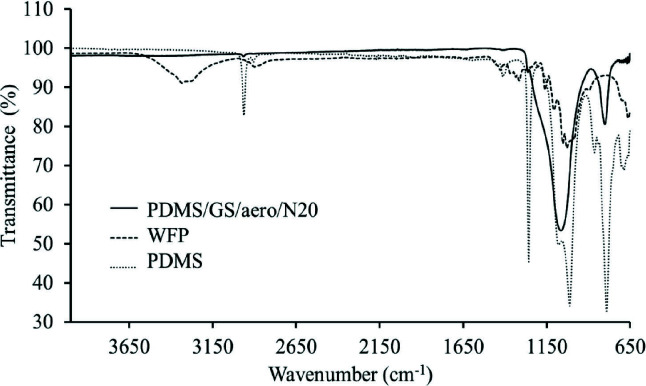
FT-IR spectra of WFP, PDMS, and PDMS/GS/aero/N20

In this study we examined specific regions in FT-IR spectra of WFP, PDMS, and PDMS/GS/aero/N20, where medium to strong peaks were observed. As revealed in Figure 2, FT-IR spectrum of WFP reveals the –OH stretching peaks of cellulose at 3314 and 3269 cm^–1 ^[48]. Moreover, the peak at 2858 cm^–1^ corresponds to the CH_2_ stretching [49]. Peaks at 1159 and 1108 cm^–1^ are attributed to the C-C asymmetric stretch and C-O-C glycosidic ether bands, respectively [50]. The peak centered at 1053 cm^–1^ indicates C-OH stretching of the secondary alcohol whereas the one at 1031 cm^–1^ corresponds to C-OH stretching of the primary alcohol in the cellulose backbone. And, the peak positioned at 662 cm^–1^ is for C-OH out of plane bending of cellulose units [48,50,51]. FT-IR spectrum of PDMS contains a strong peak at 1258 cm^–1^ which corresponds to the symmetric CH_3_ deformation of the PDMS backbone. Peaks positioned at 1079 and 1013 cm^–1^ indicate the asymmetric Si-O-Si stretching doublet [52]. 

Comparison of WFP and PDMS/GS/aero/N20 spectra revealed changes on paper substrate surface after thw coating application. In the hybrid substrates the –OH stretching peaks at 3314, 3269, 1031, and 662 cm^–1^ disappear, which was expected when Raman studies were considered. The peak at 2964 cm^–1^ correponds to the CH_2_ stretching of the PDMS backbone present in the coating layer. The peak positioned at 1248 cm^–1^ belongs to cellulose and is reported to be a combination of C-C, C-O, and C=O stretches. The broad peak at 1061 cm^–1^ is a combination of C-O-C stretch of cellulose, Si-O-Si stretch of PDMS units, and Si-O-Si of aero and N20 inorganic particles [53,54]. Moreover, the freshly formed Si-O-C bonds are thought to contribute, the presence of which was already revealed by Raman studies in our previous report. Previously, additional detailed SEM studies also revealed the durability of the covalently bonded coating layer [12].

### 3.7. Conclusion

In the present study, we prepared superhydrophobic hybrid paper sheets with durable coating layers having different formulations. The composite coating layers were composed of PDMS and different inorganic fillers, the change in which resulted in variations in the air and water barrier properties, surface roughness, and mechanical properties of the hybrid sheets. All the hybrid sheets were superhydrophobic and possessed decreased air and water barrier properties and water imbibition values with respect to those of WFP. It was seen that especially the Cobb values of the hybrid paper sheets with platelet shapes filler particles in the coating were less than the others. These samples also revealed highest air barrier properties. Moreover, the hybrid sheets gained significant mechanical strength in wet state. Thus, hybrid sheets and especially those prepareed by PDMS/MMT/K/N20 coating formulation can be good alternatives for packaging materials. 

## References

[ref1] (2014). Engineering microfluidic papers: effect of fiber source and paper sheet properties on capillary-driven fluid flow. Microfluid and Nanofluid.

[ref2] (2017). Paper-based microfluidic devices: a complex, low-cost material in high-tech applications. MRS Bulletin.

[ref3] (2014). Zhang. Cellulose.

[ref4] (2019). Combining wax printing with hot embossing for the design of geometrically well-defined microfluidic papers. ACS Applied Materials & Interfaces.

[ref5] (2010). Taschenbuch der Papiertechnick.

[ref6] (2012). Triclosan-based antibacterial paper reinforced with nano-montmorillonite: a model nanocomposite for the development of new active packaging. Polymers for Advanced Technologies.

[ref7] (2013). Barrier properties created by dispersion coating. Tappi Journal.

[ref8] (2020). Determination of hydrophobic degree of paper packaging materials by a tracer-assisted headspace gas chromatography. Nordic Pulp & Paper Research Journal.

[ref9] (2019). Fabrication of food-safe superhydrophobic cellulose paper with improved moisture and air barrier properties. Carbohydrate Polymers.

[ref10] (2019). Synthesis of polymer hybrid latex polystyrene methylmethacrylate-cobutylacrylate with organo-montmorillonite as filler through miniemulsion polymerization for barrier paper application.

[ref11] (2016). Polylactide/montmorillonite hybrid latex as a barrier coating for paper applications. Polymers.

[ref12] (2018). Superhydrophobic hybrid paper sheets with Janus type wettability. ACS Applied Materials & Interfaces.

[ref13] (2018). Surface-wetting characterization using contact-angle measurements. Nature Protocols.

[ref14] (2012). Interfacial reactions and self-adhesion of polydimethylsiloxanes. Journal of Adhesion Science and Technology.

[ref15] (2012). Triclosan-based antibacterial paper reinforced with nano-montmorillonite: a model nanocomposite for the development of new active packaging. Polymers for Advanced Tecnologies.

[ref16] (2011). Rediscovering silicones: “unreactive” silicones react with inorganic surfaces. Langmuir.

[ref17] (2011). Recent advances in clay/polymer nanocomposites. Advanced Materials.

[ref18] (2017). Effects of formation and penetration properties of biodegradable montmorillonite/chitosan nanocomposite film on the barrier of package paper. Applied Clay Science.

[ref19] (2019). PVOH modified nano-kaolin as barrier coating material for food packaging application. In: AIP Conference Proceedings 2019; Malang City.

[ref20] (2019). The influence of pigment type and loading on water vapor barrier properties of paper coatings before and after folding. Progress in Organic Coatings.

[ref21] (2018). An economic-environmental analysis of selected barriercoating materials used in packaging food products: a Swedish case study. Environment, Development, and Sustainability.

[ref22] (2019). Exploring the interactions between starches, bentonites and plasticizers in sustainable barrier coatings for paper and board. Applied Clay Science.

[ref23] (2020). Preparation of SiO2 nanoparticles with adjustable size for fabrication of SiO2/PMHS ORMOSIL superhydrophobic surface on cellulose-based substrates. Progress in Organic Coatings.

[ref24] (2017). Facile preparation of hybrid microspheres for super-hydrophobic coating and oil-water separation. Chemical Engineering Journal.

[ref25] (2019). Transparent hydrophobic hybrid silica films by green and chemical surfactants. ACS Omega.

[ref26] (2019). Facile method for the preparation of superhydrophobic cellulosic paper. Applied Surface Science.

[ref27] (2019). A free-standing superhydrophobic film for highly efficient removal of water from turbine oil. Frontiers of Chemical Science and Engineering.

[ref28] (2019). Separation of oil-water via porous PMMA/SiO2 nanoparticles superhydrophobic surface. Colloid Surface.

[ref29] (2018). Facile fabrication of superhydrophobic nanocomposite coating using modified silica nanoparticles and nonfluorinated acrylic copolymer. Polymer Bulletin.

[ref30] (2018). Facile method for the hydrophobic modification of filter paper for applications in water-oil separation. Surface and Coatings Technology.

[ref31] (2019). Improving surface-wetting characterization. Materials Science.

[ref32] (2019). Uncertainties in contact angle goniometry. Soft Matter.

[ref33] (2019). PVOH modified nano-kaolin as barrier coating material for food packaging application. In: AIP Conference Proceedings 2019; Malang City.

[ref34] (2020). Studies on semi-crystalline poly lactic acid (PLA) as a hydrophobic coating material on kraft paper for imparting barrier properties in coated abrasive applications. Progress in Organic Coatings.

[ref35] (2018). Multilayer structures based on annealed electrospun biopolymer coatings of interest in water and aroma barrier fiber-based food packaging applications. Journal of Applied Polymer Science.

[ref36] (2020). Effect of polyethylene wax/soy protein-based dispersion barrier coating on the physical, mechanical, and barrier characteristics of paperboards. Journal of Coatings Technology and Research.

[ref37] (2019). and grease-resistant multilayer coating for paper-based substrate and uses of thereof. US2019/.

[ref38] (2011). Using laser speckle to measure the roughness of paper. Tappi Journal.

[ref39] (2018). Paper physics: paper and board surface roughness characterization using laser profilometry and gray level cooccurrence matrix. Nordic Pulp & Paper Research Journal.

[ref40] (2006). Comparing low coherence interferometry with conventional methods of measuring paper roughness. In: Saratov Fall Meeting.

[ref41] (2018). Printability effect on Asam Rumex exreacted papers. Oxidation Communications.

[ref42] (2016). The effect of internal application of organosilicon compounds on the hydrophobicity of recycled OCC paper. Bioresources.

[ref43] (2017). Increased water resistance of paper treated with amylose-fatty ammonium salt inclusion complexes. Industrial Crops and Products.

[ref44] (2019). Preparation of carboxymethyl cellulose from tea stalk and its use as a paper-strengthening agent. Nordic Pulp & Paper Research Journal.

[ref45] (2015). Carboxymethylated glucomannan as paper strengthening agent. Carbohydrate Polymers.

[ref46] (1997). Purity of the sacred lotus, or escape from contamination in biological surfaces. Planta.

[ref47] (2015). Preparation and characterization of monodisperse polystyrene-silica nanocomposites. Macromolecular Research.

[ref48] (2012).

[ref49] (2009). Cellulose filter paper with antibacterial activity from surface-initiated ATRP. Jornal of Macromolecular Science, Part A: Pure and Applied Chemistry.

[ref50] (2013). Identification of cellulosic fibres by FTIR spectroscopy - thread and single fibre analysis by attenuated total reflectance. Studies in Conservation.

[ref51] (2020). Smart paper transformer: New insight for enhanced catalytic efficiency and reusability of noble metal nanocatalysts. Chemical Science.

[ref52] (2011). Fumed silica filled poly(dimethylsiloxane-urea) segmented copolymers: preparation and properties. Polymer.

[ref53] (1998). A FT-IR study of the hydrolysis of tetraethylorthosilicate (TEOS). Spectroscopy Letters.

[ref54] (2001). Conversion of polycarbosilane (PCS) to SiC-based ceramic Part 1. Characterisation of PCS and curing products. Journal of Materials Science.

